# The impact of head and neck radiotherapy on the dentine-enamel junction: a systematic review

**DOI:** 10.4317/medoral.23212

**Published:** 2019-12-24

**Authors:** Jéssica Montenegro Fonseca, Cristhian Camilo Madrid Troconis, Natália Rangel Palmier, Wagner Gomes-Silva, Mariana de Pauli Paglioni, Anna Luiza Damaceno Araújo, Lady Paola Aristizábal Arboleda, Aljomar José Vechiato Filho, Wilfredo Alejandro González-Arriagada, Mario Fernando de Goes, Márcio Ajudarte Lopes, Thaís Bianca Brandão, Pablo Agustin Vargas, Ana Carolina Prado Ribeiro

**Affiliations:** 1 Oral Diagnosis Department, Semiology and Oral Pathology Areas, Piracicaba Dental School, University of Campinas (UNICAMP), Piracicaba, São Paulo, Brazil; 2 Department of Restorative Dentistry, Piracicaba Dental School, University of Campinas (UNICAMP) Piracicaba, São Paulo, Brazil; 3 Dental Oncology Service, Instituto do Câncer do Estado de São Paulo (ICESP), Faculdade de Medicina da Universidade de São Paulo, São Paulo, Brazil; 4 Facultad de Odontología, Universidad de Valparaíso, Chile; 5 Odontologia Oncológica D’or, São Paulo, Brasil

## Abstract

**Background:**

Radiotherapy is widely used in contemporary head and neck cancer treatment protocols. The ability of head and neck radiotherapy (HNRT) to cause direct radiogenic destruction to the teeth is one of the most controversial topics in the field of oral oncology. Therefore, this systematic review aimed to investigate ionising radiation as an independent factor for physical and chemical changes on the dentine-enamel junction (DEJ), a pivotal dental topography for the onset and progression of radiation-related caries (RRC) and enamel delamination.

**Material and Methods:**

Systematic searches were conducted on three databases: Scopus, MEDLINE (Via PubMed) and Embase (Elsevier). Laboratory studies evaluating the effects of simulated or in vivo HNRT on the DEJ were included. The GRADE tool adapted for in vitro studies was used to assess the methodological quality.

**Results:**

Of the 154 initially selected studies, eight met the inclusion criteria, from which five studies were graded as high quality of evidence, two studies were graded as moderate quality and one as low quality. Two studies did not demonstrate DEJ alterations following HNRT while the other six articles described several organic and inorganic changes in the DEJ of irradiated teeth samples. These radiogenic events were mostly detected through micro and nanoindentation, Raman micro-spectroscopy, confocal microscopy, Western blotting and optical coherence tomography.

**Conclusions:**

HNRT may have a negative impact on the physical and chemical aspects of the DEJ, predisposing cancer patients to RRC and enamel delamination.

** Key words:**Cancer, radiotherapy, radiation-related caries, dentin-enamel junction, systematic review.

## Introduction

Head and neck cancer (HNC) is an important public health problem throughout the world and covers approximately 10% of all malignant tumours in developed countries, occupying the sixth place among the most common malignancies. Treatment protocols often involve the combination of surgery, chemotherapy and head and neck radiotherapy (HNRT). Although considered highly effective in the locoregional control of cancer, HNRT results in a myriad of acute and chronic toxicities to non-targeted tissues including oral mucositis, hyposalivation, oral opportunistic infections, trismus, radiation-related caries (RRC) and osteoradionecrosis, among others (1,2).

RRC is one of the most significant oral toxicities of head and neck radiotherapy (HNRT), which affects up to 25% of all cancer patients subjected to radiation therapy (3). The potential of ionising radiation to directly cause harmful effects on tooth structure, favouring enamel delamination and RRC onset and progression, is highly controversial. In this context, many studies have suggested direct radiogenic damage to dentine and enamel that could lead to RRC (4-6). Conversely, other studies have linked the increased risk of RRC to the indirect effects of radiation therapy on the structure of the teeth (7-9), such as those caused by hyposalivation, oral microbiota alterations, impaired self-cleaning properties, poor oral health status of HNC patients, increased dietary intake of carbohydrates and insufficient fluoride exposure, which act in synergy to form a cluster of oral symptoms that predisposes the teeth to caries onset and rapid progression (10).

RRC lesions do not follow the conventional caries patterns of clinical development; instead, there is an initial brownish discolouration of non-cavitated enamel surfaces and the cervical region of the teeth, incisal caries and enamel wear on molar cusps. When not diagnosed and promptly treated, RRC progresses as generalised cervical caries, enamel craze lines and cracks, enamel delamination and crown amputation, leading to diffuse dental destruction in only a few months (10). However, RRC and conventional caries are undistinguished by microscopic patterns of progression and dentine reactions to their progression (11).

Delamination is a type of failure mode for composite materials including the dentine-enamel junction (DEJ). Repeated cyclic stresses and impact can cause enamel delamination due to a biomechanical failure of dentine and inner enamel sites since they symmetrically span the DEJ, with a significant loss of mechanical toughness, leaving the exposed dentine vulnerable to subsequent decay (4,12). In addition, previously published *in vitro* studies (8,13) have suggested that direct radiogenic damage to the dentition might also impact the stability of the DEJ, leading to enamel delamination and RRC progression. 

The enamel organic matrix is mainly located in inner sites, the DEJ acts in synergy to preserve the adhesion between the enamel layer and the underlying dentine, dissipating mechanical stress between both hard-dental tissues and inhibiting further crack propagation into dentine (4,14). Clinical research suggests that the mechanical properties of the enamel are negatively affected by HNRT; however, no consensus has been established in the literature concerning the ability of ionising radiation to be directly injurious to the DEJ, increasing the risk for enamel delamination and RRC progression (15-17).

Despite controversial results regarding radiation-related damage to the DEJ microstructure in HNC patients (4,10,18), many studies aimed to evaluate that enzymatic expression favours RRC progression (9,15,19,20). Therefore, this systematic review aimed to evaluate if HNRT should be considered an independent risk factor to damage the physicochemical properties of the DEJ in cancer patients.

## Material and Methods

- Protocol and registration

This systematic review was developed according to the Preferred Reporting Items for Systematic Reviews and Meta-Analyses (PRISMA) Checklist (21). The main methodological data were previously registered at the International Prospective Register of Systematic Reviews (http://www.crd.york.ac.uk/PROSPERO/) and received the protocol number CRD42018087404.

- Eligibility Criteria 

The present study aimed to investigate the following question: “Does ionising radiation induce damage to the micromorphology properties of the DEJ contributing to caries progression?”.

To elucidate the mentioned clinical query, our search was based on *in vitro* or *in vivo* studies that analysed human teeth before and after HNRT. As established by the PRISMA guidelines, the PICO framework was designed, as follows:

1.Population: Head and neck adult cancer patients

2.Interventions: Radiation doses from 30 to 70 Grays (Gy)

3.Comparison: Irradiated teeth versus non-irradiated human teeth

4.Primary outcome: To evaluate the micromorphology property changes of DEJ

5.Secondary outcome: Metalloproteinases related to caries progression 

6.Exclusion criteria: Animal studies; studies designed to evaluate the effects of radiotherapy on independent tooth tissues (enamel, dentin, pulp and cementum) structure; total radiation doses lower than 30 Gy; comprehensive reviews, editor letters, personal opinions, book chapters, conference

abstracts and patents. 

- Search Strategy 

Electronic searches were carried out on Scopus, MEDLINE/PubMed and Embase (Elsevier) using the following strategies (adapted for each database): (‘enamel-dentine junction’ OR ‘dentine-enamel junction’) AND (‘radiation therapy’ OR ‘radiation’ OR ‘ionising radiation exposure’ OR ‘ionising radiation’ OR ‘radiation dose’ OR ‘radiotherapy’). Articles were searched until the 19th of March, 2018. In addition, the reference lists of the selected articles were hand screened to identify potentially relevant studies that could have been missed during initial electronic database searches.

- Study selection

The study selection was completed in two phases. In phase one, two authors (JMF and ARSS) independently reviewed the titles and abstracts of all the references. They selected articles that met the inclusion criteria based on their titles and abstracts. In cases of disagreement, a third author (CCMT) intervened. Studies that clearly failed the inclusion criteria were discarded, and those whose abstracts did not contain all the information needed were entered into phase two. In phase two, full articles were read to determine the research in which *in vitro* or *in vivo* studies evaluated the effects of HNRT on the DEJ. Two authors (JMF and ARSS) independently participated in phase two. The final selection was always based on the full text. The reference lists for all the included articles were critically assessed by JMF for the new articles.

- Data extraction

One author (JMF) collected the following information from the included articles: author/year, country, study design, sample size, tooth, control, assays, radiation dose, radiotherapy modality, storage solution, time storage, results and main conclusions (Table 1).

**Table 1:**
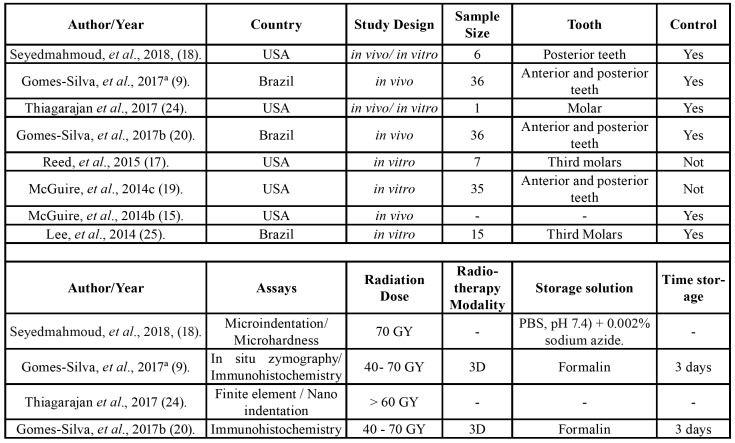
Characteristics of included studies.

**Table 1 cont.**
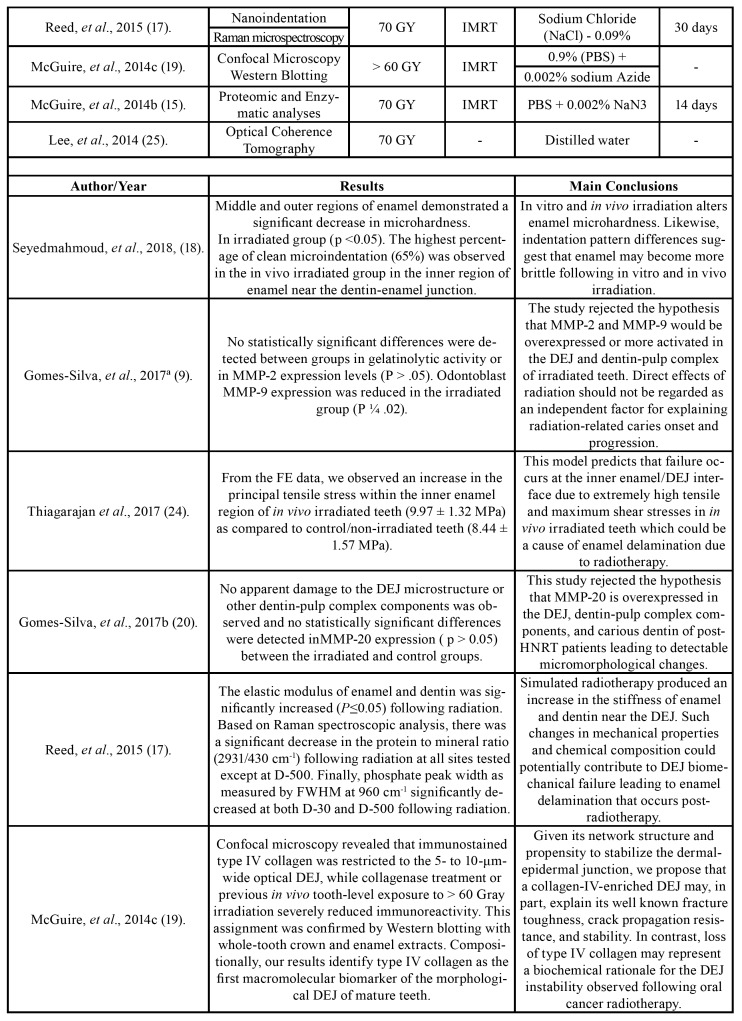
Characteristics of included studies.

**Table 1 cont.**
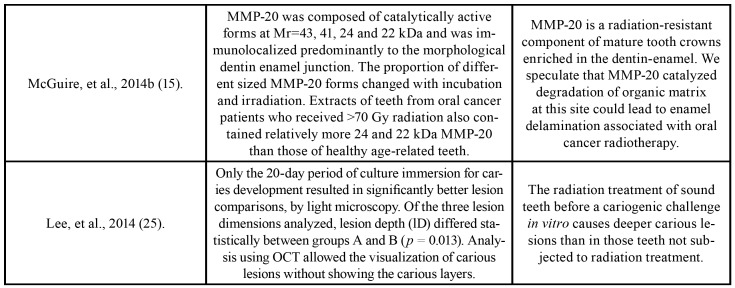
Characteristics of included studies.

**Figure 1:**
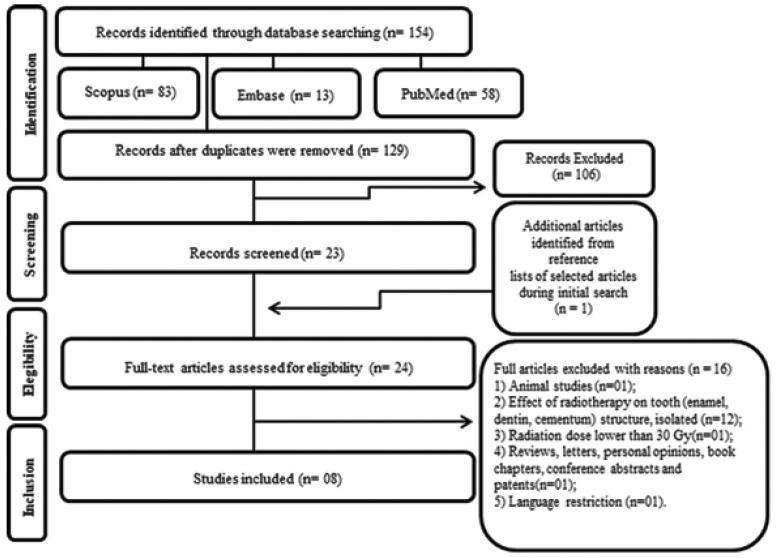
Flow Diagram of literature search and selection criteria adapted from PRISMA.

A second author (ARSS) cross-checked the collected information and confirmed its veracity. Any disagreement was resolved by a discussion and mutual agreement. Fig. 1 shows the search strategy of the selected studies obtained in the reviewing process.

- Risk of bias in individual studies

The risk of bias in the individual studies was assessed in accordance with the GRADE tool (22) for *in vitro* studies. The GRADE tool was adapted to *in vitro* studies, according to Pavan *et al*. (23), given that no specific quality assessment method was developed for this type of study. The domains below were considered.

For *in vitro* studies, two authors (JMF and ARSS) categorised the articles as ‘high’, ‘moderate’, ‘low’ or ‘very low’ overall quality of evidence, according to their analysis of each study. When they did not reach a consensus, a third author (CCMT) intervened to make a final decision.

- Summary measures

Effects of *in vitro* and *in vivo* HNRT on the microhardness, nanomechanical properties, indentation pattern and the micromorphological patterns, as well as the expression or activation of matrix metalloproteinases in the DEJ, were the main evaluated outcomes.

Due to methodological differences within the studies, a meta-analysis was inappropriate, but a detailed qualitative synthesis of the results was conducted.

## Results

Overall, 154 articles were identified from databases; after duplicate removal, 129 articles remained. A comprehensive evaluation of the titles and abstracts resulted in the exclusion of 106 articles. A full-text review was conducted on 23 articles retrieved, and an additional article was identified by reading the reference lists of these selected studies. This process led to the exclusion of 16 studies. In the end, eight articles were maintained for the final analyses (9,15,17-20,24,25). A flow diagram detailing the selection process of the study is shown in Fig. 1.

- Study characteristics

The studies were conducted in two different countries: Brazil (n=3) and the United States (n=5). All the studies were published in English from 2013 to 2018; they were divided according to the radiotherapy modality: *in vitro* (n=3), *in vivo* (n = 3) or both (n = 2). All the studies evaluated the effect of radiotherapy on the microhardness and nanomechanical properties, indentation patterns, microstructure and morphological alterations in the DEJ area, as well as the expression and activation of the matrix metalloproteinases. For these evaluations, techniques such as immunohistochemistry, micro-indentation, Raman spectroscopy, confocal microscopy, in situ zymography, finite elements, optical coherence tomography, proteomic and enzymatic analyses were used. A summary of the descriptive characteristics as well the main results and conclusions of the included studies are presented in Table 1.

- Risk of bias in individual studies

When assessed with GRADE, as seen in Table 2, five studies were graded as high quality of evidence (9,17-20); two studies were graded as moderate quality (15,25) and one as low quality (24).

Two studies presented limitations as the sample size was not reported or the study had a limited size sample (15,24). Two presented serious indirectness because of the indirectness of methods and consequently of the outcomes (24,25).

In two other studies, there was no important information regarding the characterisation of the sample including the mean radiation dose delivered; it also failed to provide data concerning the time of storage and storage solution, resulting in imprecise outcomes (15,24). Generally, the studies presented a high quality of evidence and, as a consequence, a low risk of bias.

- Synthesis of results

One study demonstrated through a microhardness test that the inner enamel (50 μm from DEJ - inner enamel and 200 μm from DEJ - middle enamel) presented decreased microhardness values following *in vitro* and *in vivo* irradiation as compared to the control group; however, this reduction was significant only (*p* < 0.05) in the middle enamel (200 μm from DEJ). The percentage of ‘clean micro-indentation’ patterns was also significantly higher in all the enamel regions close to DEJ of the irradiated group when compared to the control samples (18).

**Table 2:**
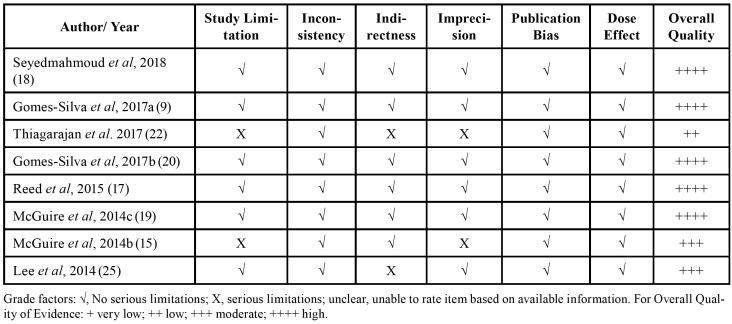
Risk of bias in individual studies. Fulfilled GRADE criteria.

Reed *et al*. (17) demonstrated that the elastic modulus of the enamel and dentin surrounding the DEJ was significantly increased (*p* ≤ 0.05) following radiation. Based on the Raman spectroscopic analysis, there was a significant decrease in the protein to mineral ratio (2931/430 cm-1) following radiation at all the sites tested except in the dentine tissue at 500 μm away from the DEJ, while the carbonate to phosphate ratio (1070/960 cm-1) increased at 30 μm away from DEJ, in the enamel, and decreased at 500 μm away from the DEJ, in the dentine. Finally, the phosphate peak width as measured at 960 cm-1 significantly decreased at both 30 μm and 500 μm away from the DEJ, in the dentine tissue, following radiation (17).

Thiagarajan *et al*. (24), through the Finite Element (FE) method, observed in the DEJ an increase in the principal tensile stress of less than 10% between the control and *in vivo* irradiated teeth with no variation in the shear stress. In addition, a difference of 3.2 MPa in the principal tensile stress between the control model principal stress and the *in vivo* model principal stress was observed.

Optical coherence tomography (OCT) was used by Lee *et al*. (25) to visualise the morphological characteristics of the caries lesions formed at the DEJ. The involvement of the DEJ and marked alterations could be clearly observed as junction continuity loss, gap formation and mineral loss.

Gomes-Silva *et al*. (9) used in situ zymography to demonstrate that the gelatinolytic activity in the DEJ and adjacent sound dentine was similar between the control and *in vivo* irradiated teeth samples (*p*>0.05). They also performed immunohistochemistry and observed similar patterns of MMP-2 and MMP-9 expression along the DEJ of both the test and control teeth samples. Likewise, a second study of the same group of researchers observed that the immunolocalization and the expression patterns of MMP-20 were similar in the DEJ microstructure components of both the control and *in vivo* irradiated teeth samples (20).

The screening of irradiated teeth crown extracts using proteomic and enzymatic analyses demonstrated that MMP-20 is a radiation-resistant component of mature tooth crowns, which is enriched in the DEJ. MMP-20 catalysed the degradation of the enamel organic matrix following radiotherapy in cancer patients, which could lead to enamel delamination associated with HNRT (15).

Confocal microscopy and Western blotting revealed that immune-stained type IV collagen at the DEJ (5 to 10 μm in width) irradiated *in vivo* presented a severe reduction in its immunoreactivity (19), suggesting that HNRT directly degrades this essential component of the organic dentine matrix.

The synthesis of the quantitative results of the included studies is presented in Table 3.

## Discussion

This systematic review included studies that evaluated the impact of HNRT on DEJ. Most of the studies did not present accurate sample characterisation (sample size, the anatomic origin of extracted teeth and modality of radiotherapy). Instead, they described through simulated or *in vivo* radiotherapy, the mechanical, physical and chemical aspects of the DEJ, which may predispose cancer patients to RRC.

Although there is a lack of literature regarding the direct radiogenic damage to dentition, the results of this systematic review suggest that HNRT may act as an independent risk factor to impact the micromorphological and biochemical features of the DEJ. One included study reported a decrease in the enamel microhardness in a region of the DEJ following *in vivo* and *in vitro* irradiation (18). In addition, another study found a significant increase in elastic modulus after simulated oral cancer radiotherapy at the evaluated sites in enamel and dentin, near the DEJ region (17); this was in agreement with two *in vitro* studies (26,27) that were not included, but in discordance with two other authors that did not observe any dental microhardness changes after *in vitro* irradiation (12,28). This divergence in the literature might be explained by the fact that the aforementioned studies did not evaluate the specific region of the DEJ.

Furthermore, a third included study (24) suggested that post-HNRT dentition failure appears to be in the inner enamel near the DEJ, not specifically at the DEJ. This can be explained by the fact that there were not any apparent differences in either the tensile and shear stress between the control and *in vitro* radiation group probably due to the observation that *in vitro* radiation increased the elastic modulus of all the regions of enamel including the DEJ.

Another issue under discussion is the use of the OCT for the carious evaluation. Lee *et al*. (25) associated the optical coherence tomography with optical light microscopy analysis and found that the irradiated tooth lesions close to the DEJ proved to be deeper than those on non-irradiated teeth. This tooth crown location (DEJ) has been chosen for the development of the lesions according to previous studies that showed a high clinical incidence of carious lesions in this dental area in irradiated head and neck patients (29,30).

The analysis assumed that the OCT method was not conclusive because it was difficult to observe the differences among the carious lesions, probably because the images of the lesions appeared somewhat tenuous and, for this reason, were not clear enough to make a comparison (25,29). Due to these difficulties in interpretation, common light microscopy analysis associated with the measurements of the carious lesions was added to this study in order to obtain quantitative data that allowed a more objective comparison (25).

**Table 3:**
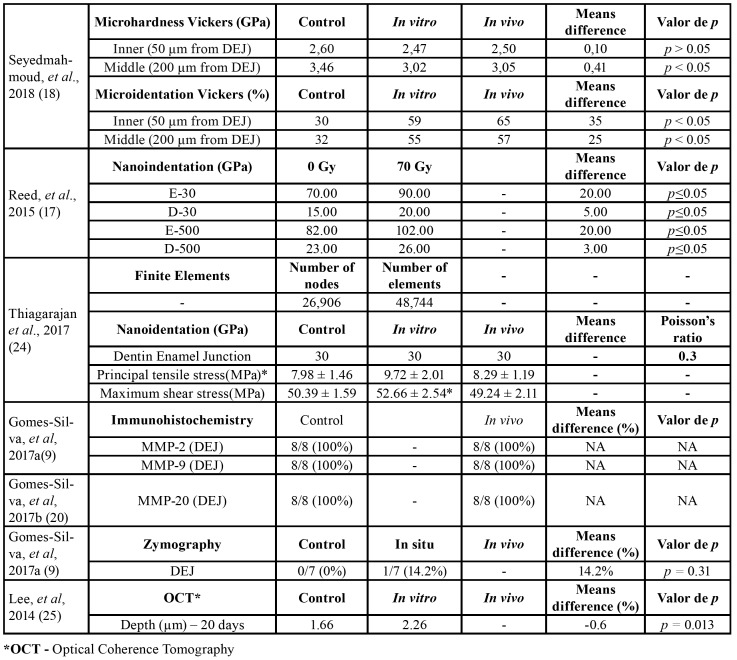
Synthesis of quantitative results.

In terms of the structural aspects of the DEJ, one study (19) included in this systematic review has focused, demonstrating (by confocal immunofluorescent staining and by Western blot analyses) type IV collagen as a DEJ component. Besides this, a reduction in DEJ collagen IV immunostaining, in *in vivo* irradiated teeth, that manifest DEJ instability, suggests that it may contribute functionally to uniting enamel to dentine. Thus, the type IV collagen may be destabilised following oral cancer radiotherapy, resulting in pathologic enamel loss followed by the decay of the exposed dentin (19,31,32).

McGuire *et al*. (19) hypothesised that *in vivo* HNRT using high doses of radiation causes induction and activation of enzymes that degrade collagens over a period of months/years and might increase the expression and activation of matrix metalloproteinases (MMPs) in various tissues (31,33), such as MMP-20, a type IV collagenase localised in the DEJ which would have its processing altered in post-radiotherapy teeth. These authors concluded that type IV collagen is a novel biomarker of the DEJ in mature human teeth and its loss following *in vivo* radiotherapy may represent a mechanism of post-irradiation DEJ instability observed in oral cancer patients, which leads to enamel delamination and dentition breakdown.

In contrast to these conclusions, another included study (20), which carried out the immunohistochemical expression of MMP-20 in post-HNRT teeth, showed that overexpression could not be observed. However, this study did not necessarily contradict the previous results of Mc-Guire *et al*. (19), as the radiation could likely affect MMP-20 activity without considerably changing the total protein amount, which cannot be detected by conventional immunohistochemical techniques.

Regarding dentine metalloproteinases, one of the studies evaluated the effects of the immunolocalisation and gelatinolytic activity of MMP-2 and MMP-9 on teeth after radiation treatment and suggested that the gelatinases MMP-2 and MMP-9 were preserved in the components of post-radiation human teeth from patients with HNC, including the DEJ (9). During the later stage of teeth formation, MMP-2 and MMP-9 are more concentrated close to the DEJ and along the mantle dentine, whereas TIMP-1 and TIMP-2, which are natural inhibitors of MMPs, are variably distributed (32,34). For this reason, the gelatinases may be constantly inhibited in the DEJ and are not directly affected by radiation.

Additional studies with well-designed methodologies are necessary to investigate the effects of ionising radiation as an independent risk factor for physical and chemical changes of the DEJ, a pivotal dental topography for the onset and progression of RRC and enamel delamination.

## Conclusions

This systematic review resulted in a small number of studies, which presented methodological heterogeneity that suggests that radiation therapy acts as an independent risk factor in causing direct radiogenic damage to the organic and inorganic components of the DEJ. Hence, well-designed methodologies, preferably longitudinal clinical studies, are necessary to identify the role of HNRT in the physicochemical properties of the DEJ in cancer patients and its potential impact on the aetiology of RRC and enamel delamination.
